# Regional Arboreal Pollen Input and Its Millennial-Scale Variability Recorded in an Alpine Lake in the Hengduan Mountains, China

**DOI:** 10.3390/biology15211995

**Published:** 2026-11-04

**Authors:** Jing Wu, Changdi Ke, Yi Huang, Hongwei Meng, Lingyang Kong, Min Wang, Qifa Sun, Caiming Shen

**Affiliations:** 1 State Key Laboratory of Lithospheric and Environmental Coevolution, Institute of Geology and Geophysics, Chinese Academy of Sciences, Beijing 100029, China; wujing@mail.iggcas.ac.cn (J.W.); huangyi@mail.iggcas.ac.cn (Y.H.); 2 Yunnan Key Laboratory of Plateau Geographical Processes and Environmental Changes, Faculty of Geography, Yunnan Normal University, Kunming 650500, China; 3 College of Earth and Planetary Sciences, University of Chinese Academy of Sciences, Beijing 100049, China; 4 School of Chemistry and Geographical Sciences, Chuxiong Normal University, Chuxiong 675000, China; wang_min@ynnu.edu.cn (M.W.); sunqifa@cxtc.edu.cn (Q.S.)

**Keywords:** Hengduan Mountains, alpine lake, pollen concentration, Asian summer monsoon, pollen transport, Holocene, millennial-scale oscillations

## Abstract

The Hengduan Mountains are a major hotspot for lake-sediment research, but regional vegetation responses to Holocene climate variability remain incompletely understood. Here, we analyzed pollen assemblages and concentrations from Dinggongniang Co (4633 m a.s.l.) to investigate regional vegetation-related changes over the past approximately 4.7 ka. Pollen percentage and concentration analyses were combined with ensemble empirical mode decomposition (EEMD) and Lomb–Scargle spectral analysis. Despite being located above the modern tree line, the lake sediments are dominated by arboreal pollen (89.5%), particularly *Pinus*, indicating substantial pollen input from lower-elevation forests. Total pollen concentrations were relatively low around 4.2 ka, increased markedly during the Roman Warm Period, and remained relatively elevated during the Medieval Climate Anomaly, suggesting changes in regional arboreal pollen input potentially associated with Asian summer monsoon-related hydroclimatic variability. Furthermore, time-series analyses identified a possible millennial-scale oscillation at approximately 1.3–1.4 ka, broadly consistent with variability documented in North Atlantic and Asian monsoon records. This finding highlights the value of high-altitude lake pollen records for tracing regional arboreal pollen input and suggests possible connections between Asian monsoon variability and broader Northern Hemisphere climate fluctuations. Although chronological uncertainties and multiple factors influencing pollen concentrations introduce some limitations, the observed patterns offer valuable insights into long-term vegetation-related environmental variability in the Hengduan Mountains.

## Simple Summary

The Hengduan Mountains in southwestern China are home to many kinds of plants, making them an important region for protecting biodiversity. However, little is known about how their forests have changed in response to past climate changes. To explore this question, we studied pollen preserved in the lake sediments of Dinggongniang Co, a mountain lake located 4633 m above sea level. The lake sediments provide a record of environmental changes over the past 4700 years. Although the lake is surrounded by grasslands rather than forests, most of the pollen found in its sediments comes from trees growing farther down the mountains. Changes in the amount of pollen suggest that climate variations, particularly changes in summer rainfall and winds, may have affected the forests and the transport of pollen to the lake. We also found that these changes followed a roughly repeating pattern over a long period, with an oscillation timescale of approximately 1300–1400 years. Our findings show that mountain lakes can help us understand how forests responded to climate changes in the past. This knowledge can help us better understand and protect mountain ecosystems as the climate continues to change.

## 1. Introduction

The Hengduan Mountains (HDMs), located on the southeastern margin of the Tibetan Plateau, are recognized as one of the world’s major biodiversity hotspots, characterized by high plant species richness, endemism, and pronounced ecological diversity [1]. Their complex topography and steep elevational gradients create diverse habitats and strong environmental contrasts over relatively short distances. Plant diversity also varies markedly across the region, with the southern HDMs supporting particularly high species richness and endemism [1]. These characteristics make the HDMs an important region for investigating vegetation responses to climate change. Furthermore, understanding past vegetation dynamics and their relationships with climatic variability can provide a long-term ecological perspective for assessing future changes in mountain ecosystems and developing appropriate biodiversity conservation strategies [1,2].

Fossil pollen preserved in lake sediments provides valuable evidence for reconstructing past vegetation and environmental changes. Numerous pollen records from the HDMs have documented Holocene changes in forest composition, vegetation belts, and regional hydroclimatic conditions [2,3,4,5,6,7,8,9,10,11,12,13,14,15,16,17]. Together, these studies demonstrate the value of lake-sediment pollen records for reconstructing long-term vegetation and environmental changes in the region. However, the interpretation and comparison of these records remain challenging because of the region’s complex environmental setting. Large elevational differences, deeply incised valleys, and contrasting influences of the Indian and East Asian monsoon systems contribute to substantial spatial variability in temperature, precipitation, and vegetation responses [18]. Although recent regional syntheses have identified broadly shared vegetation changes during the Holocene, the timing, magnitude, and ecological expression of these changes vary among sites [2,9]. These differences highlight the importance of distinguishing local vegetation signals from regional pollen inputs when interpreting high-altitude lake records.

In this context, modern pollen studies have demonstrated that pollen assemblages above the tree line in the HDMs may differ considerably from the surrounding alpine vegetation. Surface pollen samples from subalpine shrublands and meadows frequently contain abundant arboreal pollen, particularly *Pinus*, transported from forests at lower elevations [19,20,21,22,23,24,25,26,27]. The Asian summer monsoon (ASM) and mountain–valley winds may jointly facilitate this upslope pollen transport [27]. Similar contributions of extra-local arboreal pollen have also been reported in surface sediments of high-elevation lakes [28,29]. Consequently, pollen assemblages from alpine lakes above the tree line may contain both local herbaceous pollen and extra-local arboreal pollen transported from lower-elevation forests, with the latter potentially contributing substantially to the regional pollen signal. Pollen concentration, in contrast, provides complementary information on the amount of pollen preserved per unit sediment volume and may help characterize changes in regional pollen input. However, pollen concentration is not a direct measure of forest cover or rainfall, as it can also be influenced by pollen production, atmospheric transport, sediment accumulation and dilution, and pollen preservation. Despite this potential, pollen concentration remains less extensively explored than pollen percentages in Holocene vegetation reconstructions from the HDMs [3,6,7,11,12,16,30]. Its potential to provide complementary information on regional forest pollen input and associated environmental changes therefore warrants further investigation. Moreover, previous pollen studies in the HDMs have documented Holocene vegetation and hydroclimatic variability [2,3,4,5,6,7,8,9,10,11,12,13,14,15,16,17], while millennial-scale fluctuations have also been reported in North Atlantic and Asian monsoon records [31,32,33,34]. However, whether comparable timescales can be identified in high-altitude lake pollen concentration records from the HDMs remains insufficiently explored.

Dinggongniang Co (DGN Co), situated at 4633 m above sea level in the southern HDMs, provides an opportunity to investigate these issues. In this study, we analyze pollen assemblages and total pollen concentrations from a sediment sequence spanning approximately the past 4.7 ka. We aim to distinguish local and extra-local pollen contributions to the lake sediments and explore their implications for regional arboreal pollen input, as well as to investigate variations in pollen concentration and their possible relationships with ASM variability. Furthermore, we examine whether the pollen concentration record contains millennial-scale variability comparable to that reported in North Atlantic and Asian monsoon records.

## 2. Materials and Methods

### 2.1. Study Area

**Figure 1:**
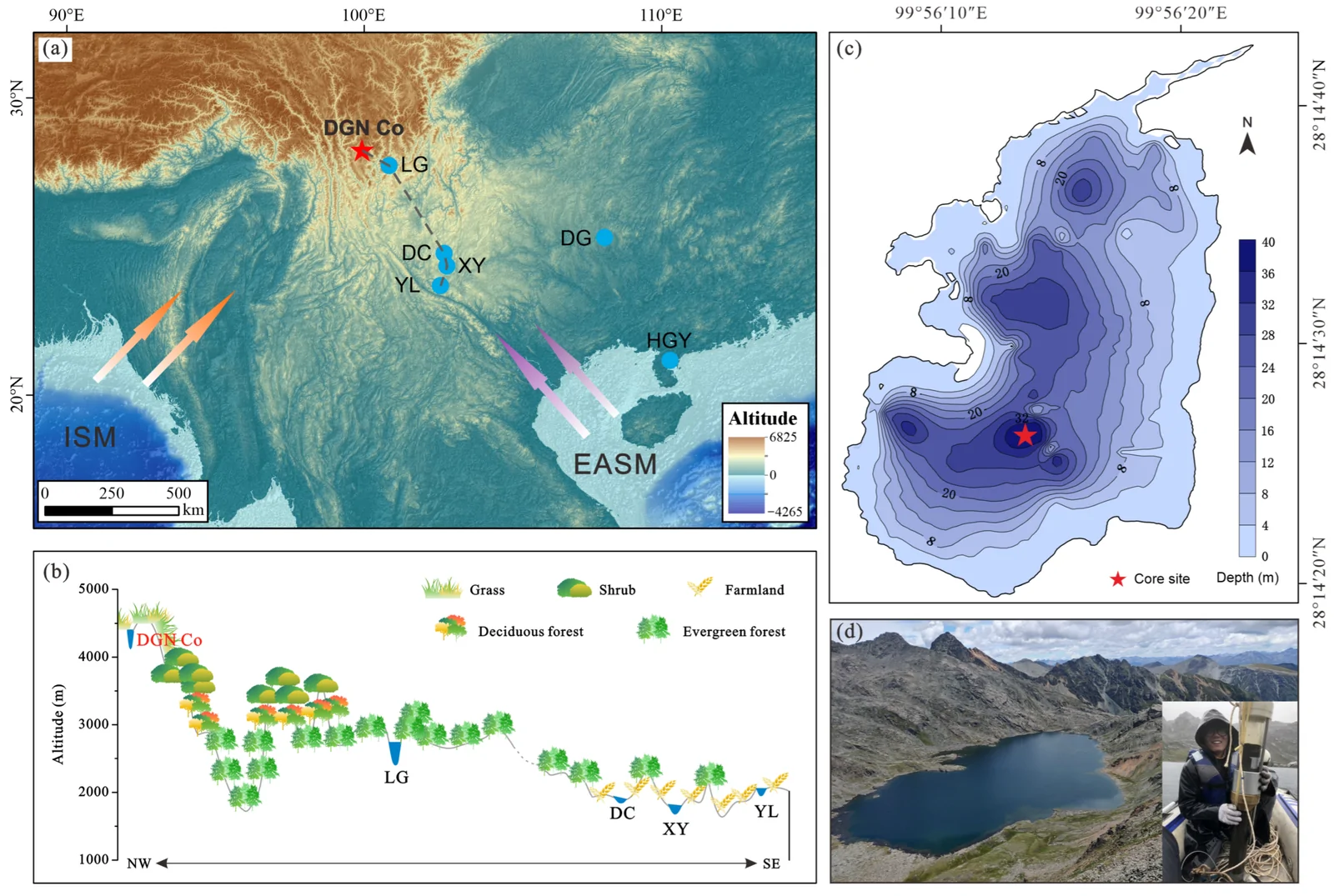
Geographical and environmental setting of Dinggongniang Co (DGN Co). (**a**) Location of DGN Co and selected lakes with pollen concentration records in the Hengduan Mountains and Asian monsoon records used for comparison, with arrows indicating the Indian summer monsoon (ISM) and East Asian summer monsoon (EASM). The bathymetric and topographic background was derived from the GEBCO_2024 Grid dataset [37]. (**b**) Schematic representation of vegetation distribution along an elevational gradient in the Hengduan Mountains. (**c**) Bathymetric map of DGN Co, with the red star marking the sediment coring site. (**d**) Photograph of DGN Co and sediment core collection. LG, Lugu Lake; DC, Dianchi Lake; XY, Xingyun Lake; YL, Yilong Lake; DG, Dongge Cave; HGY, Huguangyan Maar Lake.

DGN Co (28°14′ N, 99°56′ E; 4633 m a.s.l.) is a high-altitude lake located near the crest of the southern Shaluli Mountains, on the southeastern margin of the Tibetan Plateau (Figure 1). The Shaluli Mountains constitute a major north–south-trending mountain system within the HDMs and form the watershed between the Jinsha and Yalong Rivers. The mountain system extends approximately from 27°06′ to 30°43′ N and from 98°58′ to 102°04′ E. Its southern sector is characterized by pronounced high-mountain topography and deeply incised valley topography, producing large local relief and steep elevational gradients [35,36]. The southern HDMs are also distinguished from areas farther north by greater topographic and habitat heterogeneity and substantially higher plant diversity [1].

DGN Co occupies a high-elevation basin above the regional tree line. The lake has a surface area of approximately 0.20 km^2^, a shoreline length of approximately 2.46 km, and a maximum water depth of 39 m. It is mainly supplied by precipitation and seasonal snowmelt, with no permanent surface inflow; an outlet occurs along the northeastern shoreline. The morphology of the basin suggests a possible glacial origin. Owing to its elevation and its position near the crest of the southern Shaluli Mountains, DGN Co is surrounded predominantly by alpine vegetation rather than forest.

**Figure 2:**
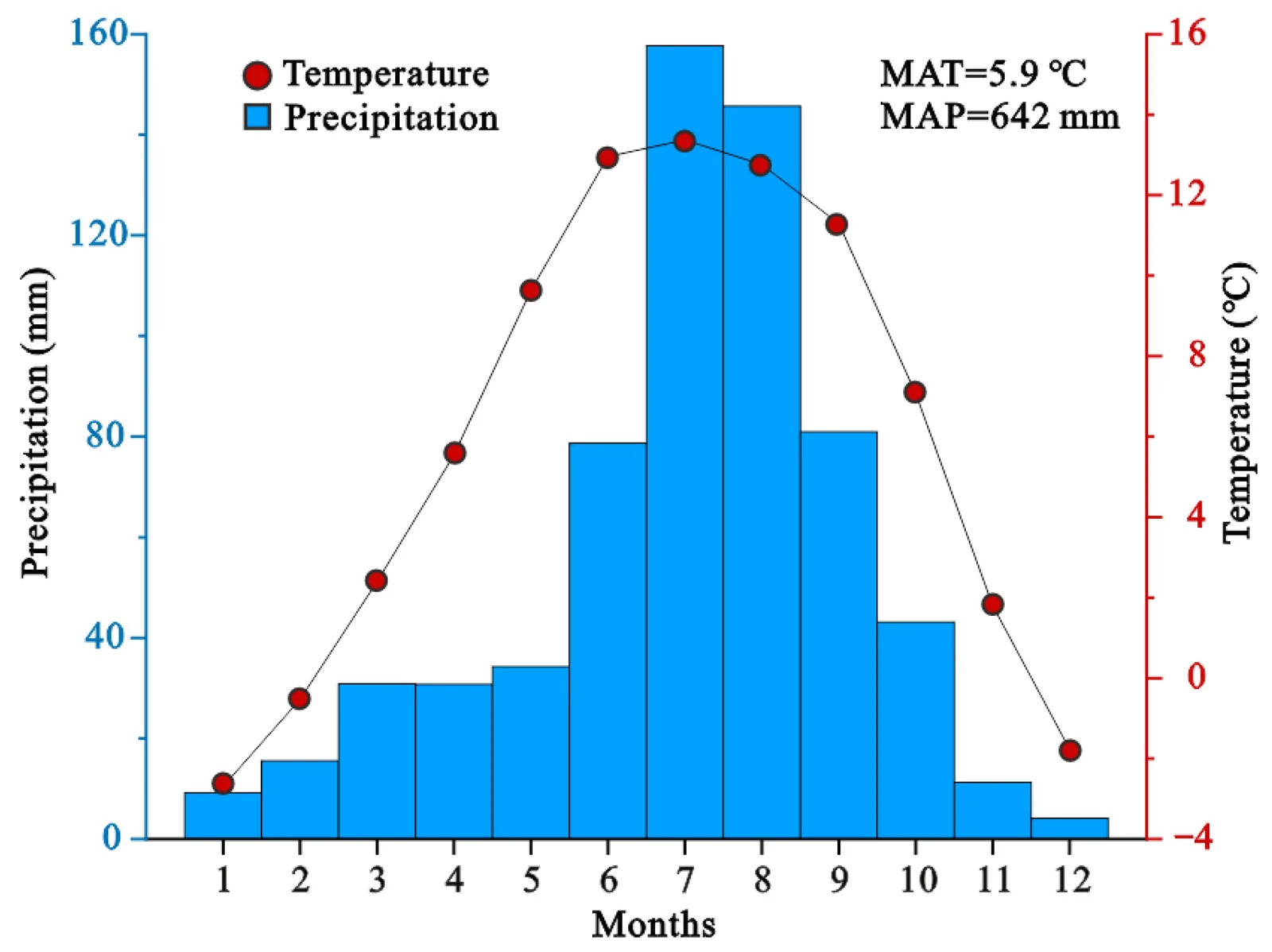
1971–2010 monthly mean precipitation and temperature at Shangri-La Meteorological Station, the closest station to DGN Co.

The regional climate is strongly influenced by the Asian monsoon system. Warm and moisture-laden air associated primarily with the ASM reaches the southeastern Tibetan Plateau during summer, whereas the winter season is dominated by cold and dry continental air masses [6]. Consequently, precipitation is therefore strongly seasonal, with most rainfall occurring during the summer monsoon season. Meteorological observations from 1971–2010 at the nearby Shangri-La Meteorological Station (27°50′ N, 99°42′ E; 3276 m a.s.l.; Figure 2) indicate a mean annual temperature of 5.9 °C and mean annual precipitation of 642 mm, with July and January being the warmest and coldest months, respectively (National Climate Center of China, China Meteorological Administration). Because the station is 1357 m lower than DGN Co, its data provide regional climatic context rather than direct estimates of lake-site conditions. Local temperature and precipitation may differ due to elevational gradients and complex topography [6], but the magnitude of these differences is difficult to quantify in the absence of site-specific observations.

Vegetation in the southern Shaluli Mountains exhibits pronounced altitudinal zonation. At lower elevations, subtropical evergreen and deciduous broadleaved forests and *Pinus*-dominated forests are widely distributed. With increasing elevation, these forests grade into montane evergreen sclerophyllous *Quercus* forests and mixed broadleaved forests containing deciduous *Quercus*, *Betula*, and *Alnus*. At higher elevations, subalpine coniferous forests are dominated primarily by *Picea* and *Abies*, with *Pinus*, *Larix*, and *Juniperus* also occurring locally. These forests are progressively replaced upward by subalpine shrublands, in which *Rhododendron*, shrubby *Quercus*, *Juniperus*, and *Salix* are important components, and subsequently by alpine meadows dominated by Cyperaceae and other alpine herbs. At the highest elevations, alpine meadows give way to sparse alpine and subnival vegetation. The elevational boundaries among these vegetation belts are not fixed and vary with local topography, slope aspect, and moisture conditions. DGN Co, at 4633 m a.s.l., lies above the regional tree line and is presently surrounded predominantly by alpine vegetation [1,6,35,36,38].

### 2.2. Coring and Dating Methods

In August 2019, a 68 cm sediment core (DGN Co core) was collected from the center of DGN Co (28°14′ N, 99°56′ E, 4633 m a.s.l.) at a water depth of 36 m (Figure 1) using a gravity corer (Pylonex AB, Umeå, Sweden). The absence of significant changes in the core lithology suggests that the depositional environment remained relatively stable. The core was sectioned with the uppermost 5 cm subsampled at 0.5 cm intervals and the remaining core subsampled at 1 cm intervals. A total of 72 sediment samples were freeze-dried prior to laboratory analysis.

Six bulk sediment samples collected at depths of 2.5, 18.5, 34.5, 50.5, 59.5, and 67.5 cm were submitted for accelerator mass spectrometry (AMS) ^14^C dating at Beta Analytic Radiocarbon Dating Laboratory. In addition, ^210^Pb and ^137^Cs activities were measured in the upper 25 cm of the core at the Yunnan Key Laboratory of Plateau Geographical Processes and Environmental Changes, Yunnan Normal University, to provide independent constraints on the recent chronology.

Excess ^210^Pb (^210^Pb_ex_) activity was determined by subtracting supported ^210^Pb, estimated from ^226^Ra activity, from total ^210^Pb activity. A preliminary chronology for the upper sediments was established using the constant rate of supply (CRS) model [39]. The ^210^Pb_ex_ inventory of each sediment interval was calculated from its activity concentration and dry sediment mass, and CRS ages were estimated from the cumulative ^210^Pb_ex_ inventory remaining below each depth. Although ^210^Pb_ex_ activity generally decreased with depth, the resulting CRS chronology was not fully consistent with the independent chronological constraints. Because the CRS model depends on an accurate estimate of the unsupported ^210^Pb inventory and assumes a constant supply of unsupported ^210^Pb, uncertainties in these conditions may have contributed to the discrepancy. However, its specific cause could not be established from the available data. The CRS-derived ages were therefore excluded from the final age–depth model.

The ^137^Cs activity profile exhibited a broad maximum across the 2.5–3.0 and 3.5–4.0 cm intervals, with no statistically distinguishable difference between their measured activities. This maximum was interpreted as corresponding to the 1963–1964 atmospheric nuclear fallout peak. The midpoint between the centers of these two intervals (3.25 cm) was therefore adopted as the representative depth of the 1964 CE marker. To assess the uncertainty associated with this assignment, sensitivity tests were conducted by placing the 1964 CE marker at depths of 2.75, 3.25, and 3.75 cm while keeping the other chronological constraints and model settings unchanged.

The six uncorrected AMS ^14^C ages showed a strong linear relationship with sediment depth (R^2^ = 0.992). Extrapolation of this relationship to the sediment surface suggested an apparent radiocarbon offset of approximately 160 ^14^C years, which was adopted as the reference reservoir correction for age–depth modelling. An uncertainty of ±30 ^14^C years was assigned with reference to the analytical uncertainties of the AMS measurements. Given the approximately linear relationship among the uncorrected radiocarbon dates, a constant reservoir correction of 160 ± 30 ^14^C years was adopted as a modelling assumption. Potential temporal changes in carbon sources, and hence the reservoir effect, cannot be excluded. The original conventional ^14^C ages and their calibrated age ranges before reservoir correction are distinguished from the reservoir-corrected ages used for modelling (Table 1). Radiocarbon ages were calibrated against the IntCal20 calibration curve [40].

The final age–depth model was constructed in R using the Bayesian age–depth modelling approach implemented in the rbacon package [41]. The core top (0 cm) was fixed to the year of core collection (2019 CE), and the ^137^Cs constraint was assigned an age of 1964 CE at 3.25 cm. The six AMS ^14^C dates were incorporated using a constant reservoir correction of delta.R = 160 and delta.STD = 30. The accumulation-rate prior (acc.mean) was set to 50 yr cm^−1^ following the ballpark estimate suggested by Bacon, while the remaining model parameters were left at their default settings. The CRS-derived ^210^Pb ages were excluded from the final model.

### 2.3. Laboratory Analysis and Numerical Methods

A total of 72 sediment samples (0.25 mL each) were processed for pollen analysis following standard chemical preparation procedures [42], including treatment with 10% HCl, 10% KOH, 40% HF, and acetolysis. One *Lycopodium* marker spore tablet (containing 9660 ± 2% exotic *Lycopodium* spores, manufactured by Lund University, batch No. 3862) was added to each sample. Pollen grains were identified and counted under an Olympus BX31 optical microscope (made by Olympus Corporation, Tokyo, Japan) at 400× magnification. Pollen identification was performed with reference to the photomicrographs provided in An Illustrated Handbook of Quaternary Pollen and Spores in China [43]. More than 500 terrestrial pollen grains were counted for each sample. Pollen percentages were calculated using the sum of terrestrial plant pollen.

Pollen concentrations were calculated using the *Lycopodium* marker method. Sediment samples were freeze-dried, and their volumes were measured using the water-displacement method. A known number of *Lycopodium* spores were added to each sample before chemical preparation. Pollen concentration (grains cm^−3^) was calculated as (P × L)/(L_(c)_ × V), where P is the number of fossil pollen grains counted, L is the number of *Lycopodium* spores added, L_(c)_ is the number of *Lycopodium* spores counted, and V is the measured volume of freeze-dried sediment (cm^3^). The pollen diagram was constructed using Tilia software (v2.6.1) [44], and pollen zones were defined based on changes in the percentages of major pollen taxa and the results of stratigraphically constrained cluster analysis (CONISS) [45].

To investigate multi-timescale variability in total pollen concentration, ensemble empirical mode decomposition (EEMD) was applied to the DGN Co pollen concentration record. EEMD is a noise-assisted, data-adaptive method that decomposes nonlinear and non-stationary time series into intrinsic mode functions (IMFs) and a residual component without requiring predefined frequency bands [46,47]. The IMFs generally represent oscillations with progressively decreasing frequencies, whereas the residual describes the slowly varying background.

The original record, comprising 72 samples spanning approximately the past 4.7 ka, was linearly interpolated onto a regular 25-year age grid from −50 to 4650 cal yr BP, yielding 189 data points. EEMD was performed using 100 complementary-noise ensemble pairs, a noise amplitude of 0.2 times the standard deviation of the input series, and a fixed random seed of 2026. The maximum number of IMFs was set to 10, yielding seven IMFs and a residual component. The internal sifting stopping conditions implemented in Acycle were used without modification [47,48]. The relative variance contribution of each component was calculated as its variance divided by the sum of the variances in all extracted components, and dominant periods were estimated from the spectral characteristics of individual IMFs. Because interpolation does not increase the independent information contained in the original 72 samples, shorter-timescale variability was interpreted cautiously.

Furthermore, to assess the dominant oscillatory timescales identified by EEMD, Lomb–Scargle spectral analysis was performed on the original 72 unevenly spaced pollen concentration observations [49,50]. A linear trend was removed before spectral analysis, while the original age coordinates were retained without interpolation. The resulting spectrum comprised 142 frequency points, spanning approximately 5.18 × 10^−5^ to 7.36 × 10^−3^ yr^−1^, with a frequency increment of approximately 5.18 × 10^−5^ yr^−1^. Spectral significance was evaluated against the robust first-order autoregressive [AR(1)] red-noise background implemented in Acycle, using a 20% median-smoothing window. The AR(1) model was used to represent temporally correlated background variability, and spectral peaks were compared with local 90%, 95%, and 99% significance thresholds. Both EEMD and Lomb–Scargle analyses were conducted using Acycle software (version 3.1.1) [48]. The identified spectral peaks were interpreted as evidence of quasi-periodic variability within the available record, rather than definitive proof of periodic climatic forcing.

## 3. Results

### 3.1. Chronology

Six AMS ^14^C dates were obtained from the DGN Co core (Table 1). The ^210^Pb_ex_ activity generally decreased with depth in the upper sediments. However, the CRS-derived chronology was not fully consistent with the independent chronological constraints and was therefore excluded from the final age–depth model. The highest ^137^Cs activities occurred in the 2.5–3.0 and 3.5–4.0 cm intervals, forming a broad maximum represented by a midpoint depth of 3.25 cm, which was assigned to 1964 CE. Sensitivity tests using alternative ^137^Cs marker depths of 2.75 and 3.75 cm produced similar age–depth models. The maximum difference in modelled median ages among the three scenarios was 41 years, while the modelled basal ages at 67.5 cm ranged from 4690 to 4701 cal a BP, indicating a limited influence of the ^137^Cs marker depth on the overall chronology.

**Figure 3:**
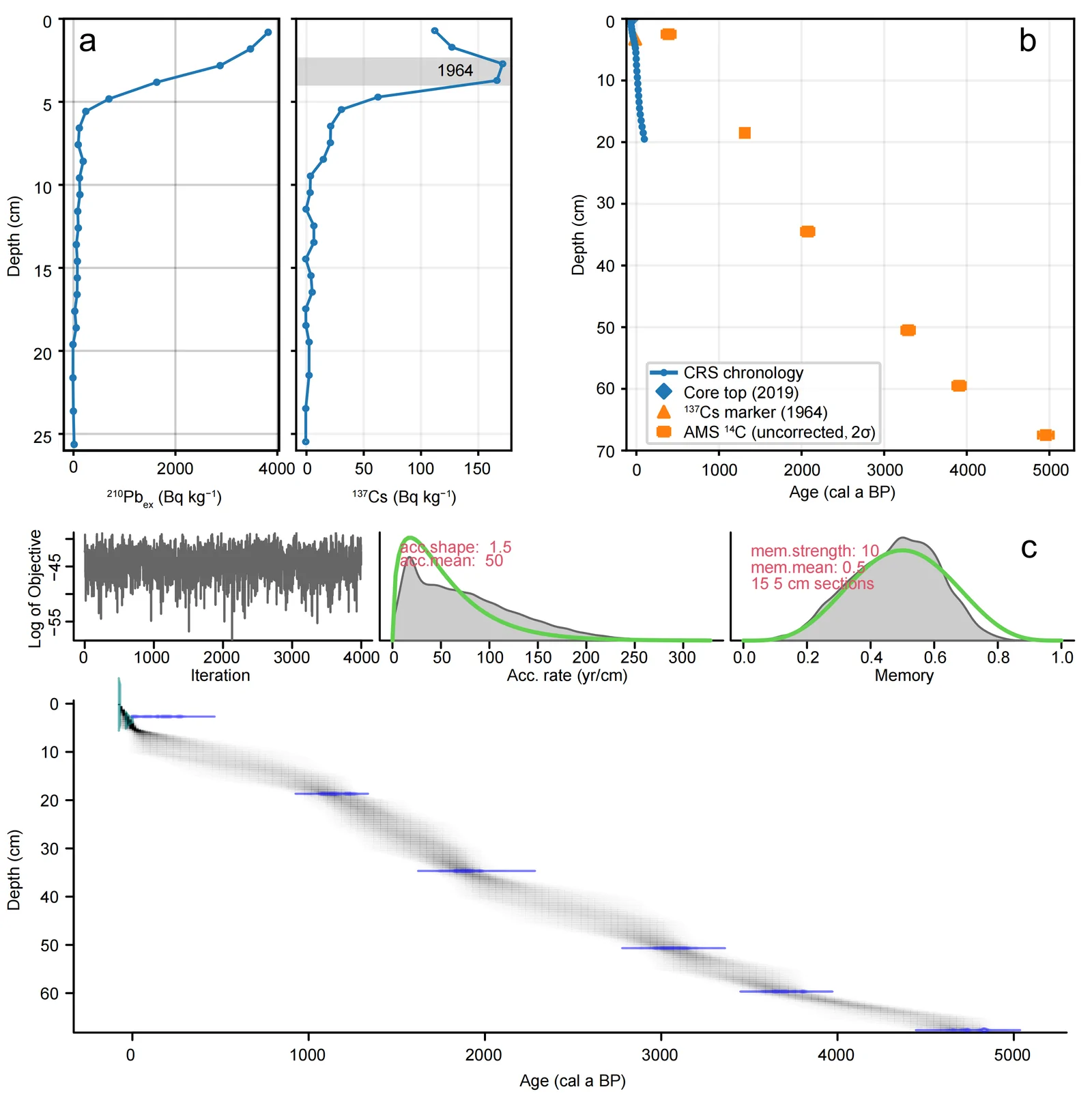
Chronology of the DGN Co sediment core. (**a**) Depth profiles of excess ^210^Pb (^210^Pb_ex_) and ^137^Cs activities in the upper 25 cm of the core. (**b**) Comparison of the ^210^Pb CRS chronology with independent chronological constraints from the top of the core, the 1964 CE ^137^Cs marker, and AMS ^14^C dates. Horizontal error bars for the AMS ^14^C dates represent 2σ calibrated age ranges prior to reservoir correction. The CRS-derived chronology was inconsistent with the independent chronological constraints and was therefore excluded from the final age–depth model. (**c**) Bayesian age–depth model constructed using Bacon based on the core-top age (2019 CE), the ^137^Cs marker (1964 CE), and six AMS ^14^C dates.

The six uncorrected bulk-sediment AMS ^14^C dates showed a strong linear relationship with sediment depth (R^2^ = 0.992). Extrapolation of this relationship to the sediment surface suggested an apparent radiocarbon offset of approximately 160 ^14^C years, which was adopted as the reference reservoir correction for age–depth modelling. Accordingly, a constant reservoir correction of 160 ± 30 ^14^C years was applied to the six AMS ^14^C dates used in the Bacon model (Figure 3). The resulting chronology spans approximately the past 4.7 ka, with a modelled median age of 4694 cal a BP at 67.5 cm.

### 3.2. Fossil Pollen Records

**Figure 4:**
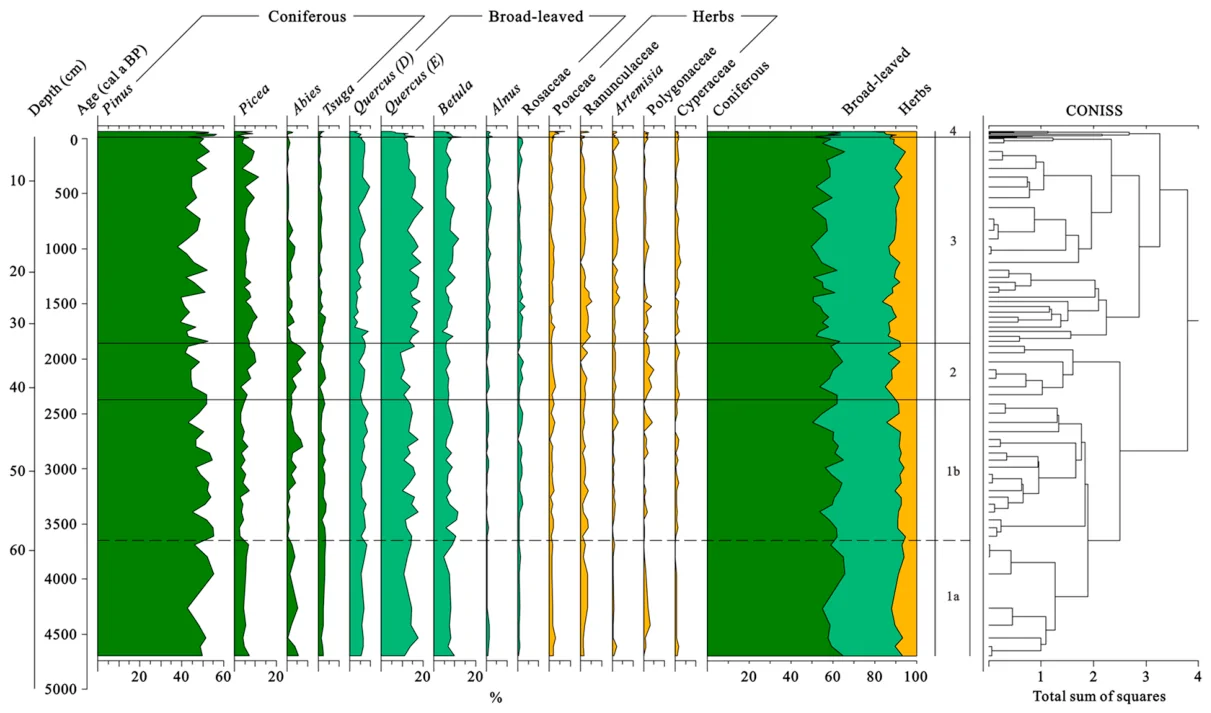
Pollen percentage diagram of selected terrestrial pollen taxa from the DGN Co sediment core, showing pollen zones and CONISS cluster analysis results.

**Figure 5:**
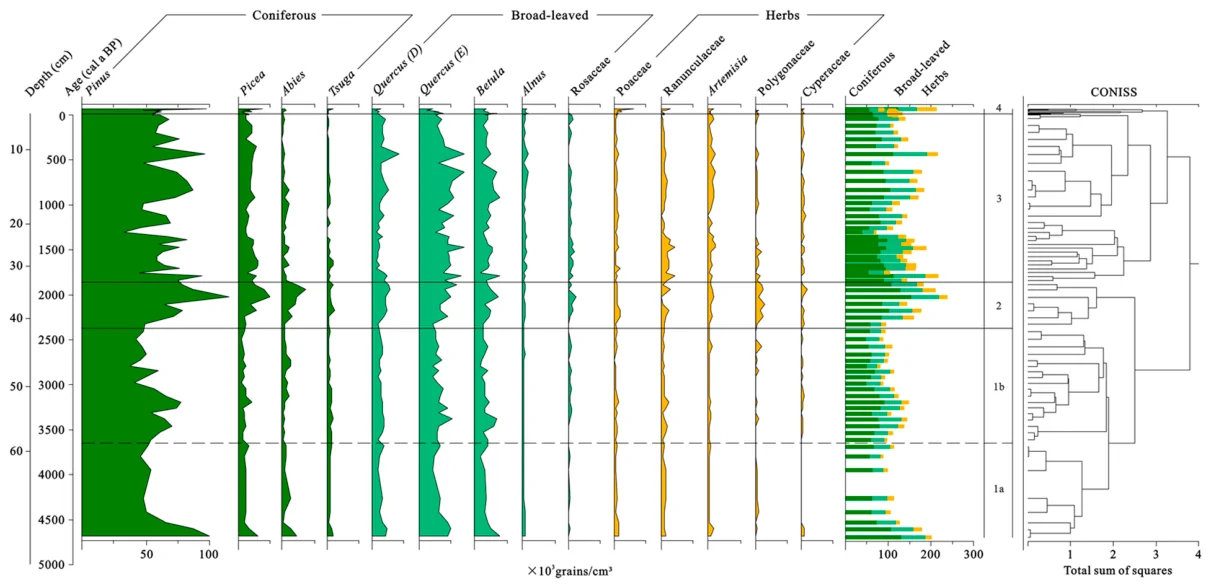
Pollen concentration diagram of selected terrestrial pollen taxa from the DGN Co sediment core, with concentrations expressed as ×10^3^ grains/cm^3^ and pollen zones based on CONISS analysis of percentage data.

Pollen spectra from the DGN Co core are dominated by arboreal pollen (AP), including coniferous and broad-leaved taxa. The main coniferous taxa are *Pinus*, *Picea*, *Abies*, and *Tsuga*, whereas broad-leaved taxa include *Quercus* (*E*), *Quercus* (*D*), *Betula*, *Alnus*, and Rosaceae. Herbaceous pollen is represented by Poaceae, Ranunculaceae, *Artemisia*, Polygonaceae, and Cyperaceae. For descriptive purposes, pollen taxa were grouped into coniferous, broad-leaved, and herbaceous categories, with broad-leaved taxa further classified as evergreen or deciduous where applicable. The distinction between evergreen and deciduous *Quercus* was made cautiously, based on pollen morphological characteristics. Based on CONISS analysis of the percentages of major pollen taxa, the 72 pollen samples from the DGN Co sediment core were divided into four pollen zones (Figure 4). The corresponding pollen concentration profiles are shown in Figure 5.

Zone 1 (68–41 cm, 4694–2327 a BP) is characterized by consistently high proportions of coniferous pollen, with *Pinus* being the most abundant taxon throughout the zone. Evergreen and deciduous broad-leaved pollen constitute the other major arboreal components, whereas herbaceous pollen occurs at relatively low percentages. Based on changes in the pollen assemblages and the CONISS results, Zone 1 is further divided into two subzones.

Zone 1a (68–59 cm, 4694–3687 a BP) is characterized by relatively high percentages of *Picea* (5.6%) and *Abies* (3.2%) within the coniferous component. Coniferous pollen averages 60.6%, dominated by *Pinus* (49.3%), with *Picea*, *Abies*, and *Tsuga* (2.6%) as secondary components. Evergreen and deciduous broad-leaved pollen average 13.6% and 17.2%, respectively, with evergreen *Quercus* (13.6%), *Betula* (7.8%), and deciduous *Quercus* (6.4%) as the major taxa. Herbaceous pollen averages 8.6%, with Ranunculaceae (2.1%) and Poaceae (1.9%) being relatively abundant.

The mean total pollen concentration is 128,054 grains/cm^3^. Coniferous pollen has a mean concentration of 77,722 grains/cm^3^, dominated by *Pinus* (62,976 grains/cm^3^), with *Picea* (7205 grains/cm^3^), *Abies* (4327 grains/cm^3^), and *Tsuga* (3144 grains/cm^3^) as secondary contributors. The mean concentration of broad-leaved pollen is 39,264 grains/cm^3^, with evergreen *Quercus*, *Betula*, and deciduous *Quercus* averaging 17,237, 10,168, and 8146 grains/cm^3^, respectively. Herbaceous pollen has a mean concentration of 11,044 grains/cm^3^, with Ranunculaceae and Poaceae averaging 2511 and 2481 grains/cm^3^, respectively.

Zone 1b (59–41 cm, 3612–2327 a BP) is distinguished from Zone 1a mainly by a decrease in *Picea* from 5.6% to 4.2%, accompanied by a slight decrease in *Abies* from 3.2% to 2.6%, whereas *Pinus* increases from 49.3% to 50.4%. Despite these changes, the overall proportions of the major pollen groups remain relatively stable. Coniferous pollen averages 59.8%, while evergreen and deciduous broad-leaved pollen average 13.9% and 17.4%, respectively. The principal broad-leaved taxa are evergreen *Quercus* (13.8%), *Betula* (7.8%), and deciduous *Quercus* (6.7%). Herbaceous pollen remains at an average of 8.6%, mainly represented by Ranunculaceae (1.9%) and Poaceae (1.6%).

The mean total pollen concentration decreases from 128,054 grains/cm^3^ in Zone 1a to 109,385 grains/cm^3^ in Zone 1b. The mean coniferous pollen concentration decreases to 65,337 grains/cm^3^, of which *Pinus* accounts for 55,252 grains/cm^3^, followed by *Picea* (4618 grains/cm^3^), *Tsuga* (2825 grains/cm^3^), and *Abies* (2631 grains/cm^3^). Broad-leaved pollen averages 34,246 grains/cm^3^, including evergreen *Quercus* (15,153 grains/cm^3^), *Betula* (8629 grains/cm^3^), and deciduous *Quercus* (7246 grains/cm^3^). Herbaceous pollen concentration averages 9444 grains/cm^3^, with Ranunculaceae and Poaceae averaging 2149 and 1694 grains/cm^3^, respectively.

Zone 2 (41–34 cm, 2252–1885 a BP) is distinguished from Zone 1b by a marked decrease in *Pinus* from 50.4% to 44.5%, accompanied by increases in *Picea* from 4.2% to 7.3% and *Abies* from 2.6% to 5.6%. Herbaceous pollen also increases from 8.6% to 11.5%, with Polygonaceae reaching 3.0%. In contrast, evergreen broad-leaved pollen decreases from 13.9% to 11.7%, while deciduous broad-leaved pollen remains relatively stable at 17.0%. Coniferous pollen as a whole remains dominant, averaging 59.4%. In addition to *Pinus*, *Picea*, and *Abies*, *Tsuga* averages 2.1%. The major broad-leaved taxa are evergreen *Quercus* (11.6%), *Betula* (6.6%), and deciduous *Quercus* (6.5%). Among herbaceous taxa, Polygonaceae (3.0%), Poaceae (1.8%), and Ranunculaceae (1.6%) are relatively abundant.

A prominent feature of Zone 2 is the marked increase in pollen concentration. The mean total pollen concentration increases from 109,385 grains/cm^3^ in Zone 1b to 185,428 grains/cm^3^, the highest value among the four pollen zones. Coniferous pollen concentration increases to 110,951 grains/cm^3^, including *Pinus* (82,622 grains/cm^3^), *Picea* (14,146 grains/cm^3^), *Abies* (10,356 grains/cm^3^), and *Tsuga* (3758 grains/cm^3^). Similarly, broad-leaved pollen concentration increases to 52,740 grains/cm^3^, with evergreen *Quercus*, *Betula*, and deciduous *Quercus* averaging 21,179, 12,374, and 11,810 grains/cm^3^, respectively. Herbaceous pollen concentration also increases to 20,838 grains/cm^3^, with Polygonaceae and Poaceae reaching 5217 and 3207 grains/cm^3^, respectively.

Zone 3 (34–4 cm, 1839 to −4 a BP) is distinguished from Zone 2 by a decrease in coniferous pollen from 59.4% to 55.8%, particularly in *Picea* (from 7.3% to 6.9%) and *Abies* (from 5.6% to 1.5%). In contrast, evergreen broad-leaved pollen increases from 11.7% to 15.7%, mainly reflecting an increase in evergreen *Quercus* from 11.6% to 15.2%. *Pinus* increases slightly from 44.5% to 45.5%. Deciduous broad-leaved pollen remains relatively stable at 16.9%, with *Betula* and deciduous *Quercus* averaging 7.6% and 5.3%, respectively. Herbaceous pollen decreases slightly from 11.5% to 10.9%, mainly including Ranunculaceae (2.3%), *Artemisia* (1.9%), and Poaceae (1.5%).

The increase in pollen concentration observed in Zone 2 is not sustained in Zone 3, with the mean total pollen concentration decreasing to 144,034 grains/cm^3^. Coniferous pollen concentration decreases to 80,034 grains/cm^3^, dominated by *Pinus* (65,341 grains/cm^3^), followed by *Picea* (9736 grains/cm^3^) and *Abies* (2231 grains/cm^3^). Broad-leaved pollen concentration averages 47,089 grains/cm^3^, with evergreen *Quercus*, *Betula*, and deciduous *Quercus* averaging 21,970, 10,930, and 7659 grains/cm^3^, respectively. The mean herbaceous pollen concentration is 16,031 grains/cm^3^, with Ranunculaceae, *Artemisia*, and Poaceae averaging 3444, 2826, and 2108 grains/cm^3^, respectively.

Zone 4 (above 4 cm, −4 a BP to present) is most notably characterized by an increase in herbaceous pollen from 10.9% in Zone 3 to 14.2% in Zone 4, the highest mean percentage among the four pollen zones, with Poaceae increasing from 1.5% to 3.5%. At the same time, evergreen broad-leaved pollen decreases markedly from 15.7% to 10.8%, mainly due to a decrease in evergreen *Quercus* from 15.2% to 10.2%. Deciduous broad-leaved pollen also decreases slightly from 16.9% to 15.4%. Despite these changes, coniferous pollen remains the largest component and increases slightly from 55.8% to 58.1%, dominated by *Pinus*, which increases from 45.5% to 49.8%, followed by *Picea* (5.4%) and *Tsuga* (1.4%). Among the deciduous broad-leaved taxa, *Betula* and deciduous *Quercus* average 8.1% and 4.0%, respectively.

The mean total pollen concentration decreases further from 144,034 grains/cm^3^ in Zone 3 to 127,306 grains/cm^3^. Coniferous pollen has a mean concentration of 73,706 grains/cm^3^, dominated by *Pinus* (62,474 grains/cm^3^), followed by *Picea* (7369 grains/cm^3^) and *Tsuga* (1893 grains/cm^3^). Broad-leaved pollen concentration decreases to 32,741 grains/cm^3^, with evergreen *Quercus*, *Betula*, and deciduous *Quercus* averaging 12,454, 10,005, and 5073 grains/cm^3^, respectively. Herbaceous pollen has a mean concentration of 18,828 grains/cm^3^, with Poaceae reaching 4950 grains/cm^3^.

### 3.3. Multi-Timescale Variability in Total Pollen Concentration

**Figure 6:**
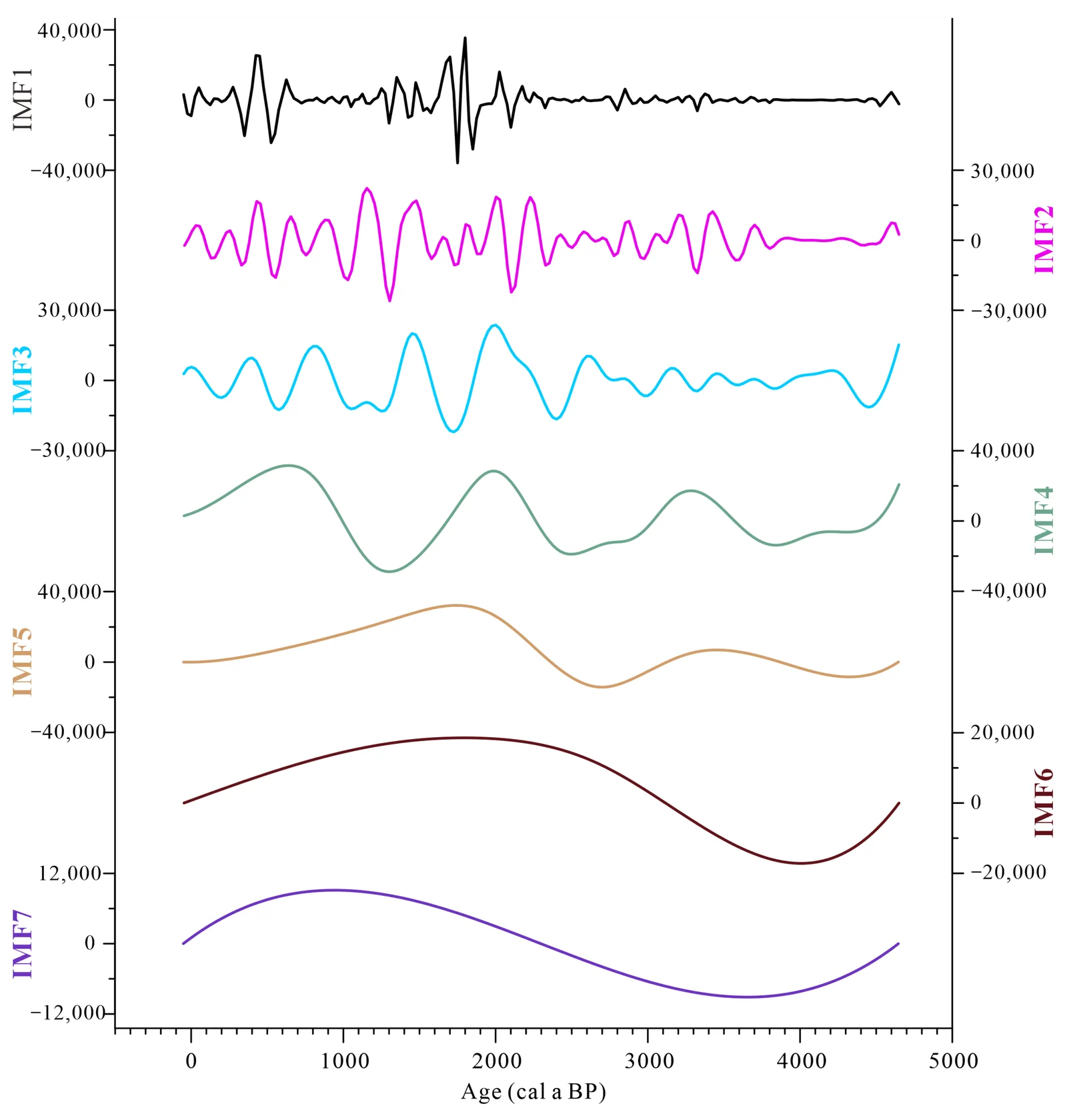
Ensemble empirical mode decomposition (EEMD) of the total pollen concentration record from DGN Co, showing seven intrinsic mode functions (IMF1–IMF7) at different timescales. The horizontal axis represents age (cal a BP), and the vertical axis represents the amplitude of each component.

EEMD analysis revealed oscillatory components with different characteristic timescales in the DGN Co total pollen concentration record (Figure 6). The decomposition of the 25-year interpolated series yielded seven intrinsic mode functions (IMF1–IMF7) and a residual component. The IMFs generally exhibited progressively lower-frequency variability with increasing mode order. IMF4 accounted for the largest proportion of the summed component variances (26.52%), followed by the residual component (17.96%), IMF5 (16.71%), and IMF6 (14.64%). The higher-frequency components IMF1–IMF3 contributed 5.27%, 6.68%, and 8.06%, respectively. IMF4 exhibited a dominant oscillation period of approximately 1.3 ka, representing the largest individual oscillatory contribution to the summed component variances. Additional fluctuations at centennial and longer timescales were distributed among the remaining IMFs.

**Figure 7:**
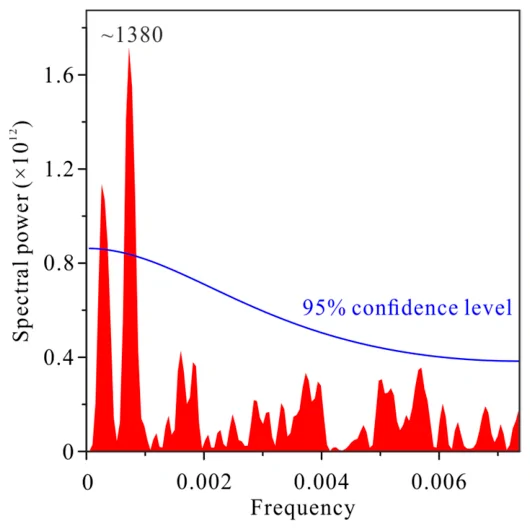
Lomb–Scargle power spectrum of the linearly detrended total pollen concentration record from DGN Co, calculated from the original 72 unevenly spaced observations. The blue line indicates the local 95% confidence threshold based on a robust AR(1) red-noise model.

To further evaluate this variability, Lomb–Scargle spectral analysis of the original 72-point, unevenly spaced total pollen concentration record revealed a prominent spectral peak at approximately 1.38 ka (Figure 7). Before detrending, this peak exceeded the local 90% significance threshold but remained below the 95% threshold of the robust AR(1) red-noise model. Following linear detrending, the peak remained at approximately 1.38 ka and exceeded the local 95% significance threshold, although it did not reach the 99% threshold. The correspondence between this spectral peak and the approximately 1.3 ka oscillatory component identified by EEMD suggests the presence of quasi-millennial-scale variability in the DGN Co total pollen concentration record. However, the statistical significance of this feature depends on detrending, and the reported significance levels refer to local rather than global spectral significance.

## 4. Discussion

### 4.1. Pollen Source and Regional Representativeness of the DGN Co Record

The pollen assemblages preserved in DGN Co sediments cannot be interpreted as a direct representation of the vegetation immediately surrounding the lake. DGN Co lies above the modern tree line and is presently surrounded by alpine meadows, yet herbaceous pollen accounts for only 10.5% of the terrestrial pollen assemblage on average, whereas arboreal pollen (AP) reaches 89.5%. The major herbaceous taxa, including Poaceae, Thalictrum, Ranunculaceae, *Artemisia*, Polygonaceae, and Cyperaceae, are common components of alpine meadow pollen assemblages in the Hengduan Mountains (HDMs). Modern vegetation surveys similarly show that alpine shrubland and meadow/tundra above ca. 4200 m are dominated by Cyperaceae, Poaceae, and Ranunculaceae [20]. Thus, the low herbaceous pollen percentage at DGN Co does not necessarily indicate sparse local herbaceous vegetation. Rather, the marked dominance of AP suggests that the pollen assemblage is influenced by sources beyond the immediate lake surroundings, potentially including forests below the modern tree line.

Modern pollen studies indicate considerable spatial variability in the representation of alpine vegetation across the HDMs. Toward the western HDMs and the adjacent Tibetan Plateau, where alpine meadow, steppe, and shrubland occupy extensive high-elevation surfaces, herbaceous pollen generally constitutes an important component of modern pollen assemblages [20]. Lake surface sediments from alpine meadows on the eastern Tibetan Plateau similarly contain abundant Cyperaceae, *Artemisia*, and Poaceae [28]. In contrast, high-elevation pollen assemblages in the eastern and southern HDMs frequently contain large proportions of arboreal pollen that may originate from forests below the tree line. This pattern has been documented in the Shadecuo profile in southwestern Sichuan [21], the Shangri-La region [26], and Yulong Snow Mountain [24,27]. For example, abundant exotic anemophilous pollen, including *Pinus* (53–90%), has been reported in subalpine scrub and meadow on Yulong Snow Mountain [24,27]. These observations suggest that above-tree-line pollen assemblages may differ spatially across the HDMs, with a stronger herbaceous component in some plateau-facing areas and a greater contribution of extra-local arboreal pollen in the deeply dissected eastern and southern mountain systems. However, these regional patterns provide a context for interpreting the DGN Co assemblage rather than direct evidence of the source or transport distance of individual pollen taxa at this site.

Evidence from lake surface sediments is particularly relevant to the interpretation of DGN Co. Unlike individual topsoil samples, lake sediments can integrate pollen from multiple locations within and beyond a lake catchment, depending on pollen production, dispersal, and depositional processes. At Xingyun Lake, for example, surface-sediment pollen assemblages are considerably more homogeneous than catchment topsoil assemblages and have been interpreted as representing regional rather than predominantly local vegetation [19]. Similarly, a regional lake-surface dataset shows that high-elevation lakes in the HDMs receive substantial amounts of arboreal pollen, particularly *Pinus*, potentially transported from vegetation at lower elevations [29]. These observations demonstrate that extra-local arboreal pollen can be an important component of lake-sediment assemblages in the HDMs. Nevertheless, the relative contributions of local and extra-local pollen depend on site-specific conditions and cannot be determined from pollen percentages alone.

Consistent with these observations, the DGN Co assemblage exhibits a similar pattern. Although the lake is surrounded by alpine meadows, AP accounts for 89.5% of the terrestrial pollen assemblage on average, with *Pinus* alone contributing 47.6%. The high AP percentage, together with observations from other high-elevation sites in the HDMs, supports the interpretation that the DGN Co record includes a substantial extra-local arboreal pollen contribution, potentially from regional forests below the tree line. Local alpine meadows are likely to contribute much of the herbaceous pollen, but the respective contributions of local and regional sources cannot be quantified using the available data. No site-specific modern pollen calibration or pollen source-and-transport modelling was conducted to determine pollen source areas or transport distances. Accordingly, changes in AP percentages should not be interpreted as direct evidence of changes in regional forest cover or productivity alone, because they may also reflect variations in pollen production, atmospheric transport, depositional processes, and the relative input of herbaceous pollen. The DGN Co pollen record is therefore interpreted as integrating local and extra-local vegetation signals, with the high AP proportion indicating a likely substantial contribution from beyond the immediate lake surroundings.

### 4.2. Asian Summer Monsoon Variability Inferred from Pollen Concentration

The amount of regional forest pollen reaching DGN Co is primarily influenced by the availability of pollen from source vegetation and the efficiency of atmospheric transport. Changes in ASM intensity may affect both processes. Enhanced summer monsoon moisture, together with favorable growing-season temperatures, can promote forest expansion and an upward shift in the upper forest belt, increase the abundance of pollen-producing trees and reduce the elevational distance between forest pollen sources and the lake. Evidence for such vegetation responses has been documented across the southeastern Tibetan Plateau and southwestern China. At Lake Naleng in the northern HDMs, *Abies*–*Betula* forests extended to higher elevations during the early and middle Holocene, when summer monsoon conditions were stronger than at present. The reconstructed tree line was approximately 400–600 m higher than today, whereas forest retreat after ~4.4 ka BP accompanied climatic deterioration [6]. Similarly, the Lake Yilong record indicates extensive forest development under warm conditions and a strengthened summer monsoon during the early Holocene [7]. These records suggest that favorable monsoon-related hydroclimatic conditions can increase regional forest cover and bring forest pollen sources closer to high-elevation lakes. At DGN Co, such changes could increase the amount of arboreal pollen available for transport from the surrounding montane forests.

Atmospheric transport provides a second mechanism linking regional forest pollen input to ASM variability. The steep elevation gradients and deeply incised valleys of the HDMs favor exchanges of air and pollen between lower forest belts and alpine environments. Modern pollen studies have documented substantial arboreal pollen deposition above the tree line, including pollen from *Pinus*, *Picea*, *Abies*, *Betula*, and evergreen *Quercus* [6,20,21]. Furthermore, previous research has suggested that the ASM may influence upslope pollen transport in the HDMs [27]. Stronger monsoon circulation could therefore favor the delivery of forest pollen to DGN Co when atmospheric and mountain–valley wind conditions are conducive to upslope transport. Together with an expanded or more proximal forest pollen source, this mechanism provides a plausible explanation for increased arboreal pollen input during periods of enhanced ASM influence. Although pollen concentration is also affected by sediment accumulation, pollen preservation, and catchment-derived inputs, its variations at DGN Co may retain a regional hydroclimatic signal associated with ASM variability.

**Figure 8:**
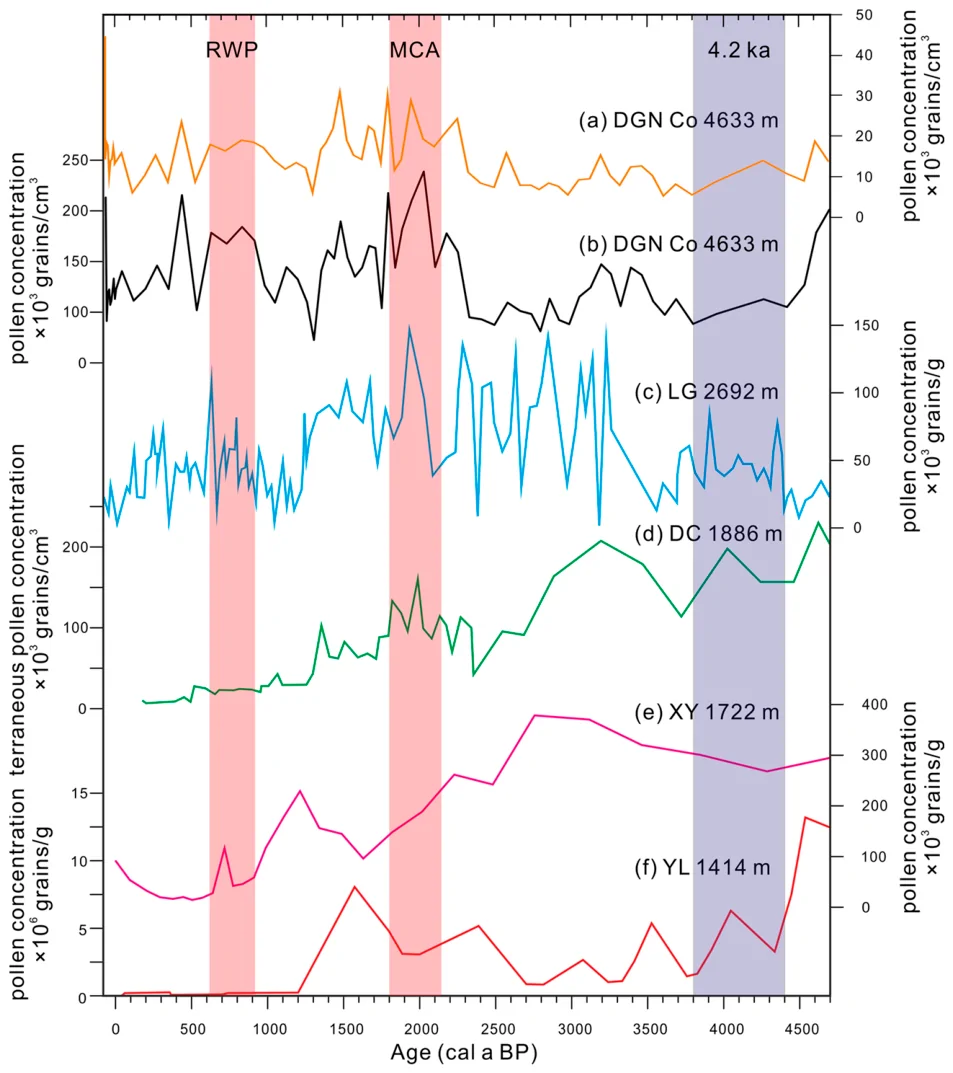
Comparison of pollen concentration records from DGN Co and selected lakes in southwestern China over the past ~4.6 ka. (a) Herbaceous pollen concentration at DGN Co (4633 m; this study); (b) total pollen concentration at DGN Co (4633 m; this study); (c) total pollen concentration at Lugu Lake (LG; 2692 m) [30]; (d) total pollen concentration at Dianchi Lake (DC; 1886 m) [16]; (e) total pollen concentration at Xingyun Lake (XY; 1722 m) [3]; and (f) total pollen concentration at Yilong Lake (YL; 1414 m) [7]. Red shading indicates the Roman Warm Period (RWP) and Medieval Climate Anomaly (MCA), while blue shading marks the 4.2 ka event.

A comparison with pollen concentration records from other lakes in southwestern China provides further support for a possible ASM-related signal at DGN Co, although the correspondence varies among sites (Figure 8). Around the 4.2 ka event, relatively low pollen concentrations are observed at DGN Co, Lugu Lake [30], and Yilong Lake [7] (Figure 8(b,c,f)). During the interval conventionally referred to as the Roman Warm Period, total pollen concentration at DGN Co reaches its highest values, while relatively high concentrations also occur at Lugu Lake [30] and Dianchi Lake [16] (Figure 8(b–d)). This correspondence may reflect favorable regional hydroclimatic conditions, although the Roman Warm Period cannot be assumed to represent a uniformly strengthened ASM across southwestern China. During the Medieval Climate Anomaly (MCA), pollen concentrations are again relatively high at DGN Co and Lugu Lake [30] (Figure 8(b,c)). In contrast, changes in pollen concentration are less pronounced at the lower-elevation Dianchi [16], Xingyun [3], and Yilong lakes [7] (Figure 8(d–f)), although a modest peak at Xingyun Lake may indicate wetter conditions in parts of southwestern China during this period. These differences may partly reflect increasing human disturbance in the lowland lake catchments. For example, cultivation around Dianchi became evident by ~2.4 ka BP [16], while the Xingyun Lake catchment experienced increasing land use and soil erosion during the past two millennia, particularly after ~750 CE [19,51]. Similarly, at Yilong Lake, human activities increasingly influenced vegetation changes during the past ~3 ka [7]. Such disturbances may have altered forest cover, pollen production, and sediment delivery, complicating the climatic interpretation of pollen concentration in these lakes. At DGN Co, the increase in total pollen concentration during the MCA is not accompanied by a comparable rise in herbaceous pollen concentration (Figure 8(a,b)), indicating that non-herbaceous pollen accounts for most of the increase. The contrast between DGN Co and the lower-elevation lakes suggests that human disturbance may have increasingly modified the pollen concentration–climate relationship in some lowland catchments, whereas DGN Co may have retained a stronger signal of regional forest pollen input associated with ASM-related hydroclimatic variability. Nevertheless, differences among the lake records may also reflect variations in pollen source areas and depositional conditions, as well as differences in measurement units, sampling resolution, and chronological uncertainty. Therefore, the records are compared primarily in terms of broad temporal patterns rather than absolute pollen concentrations. Overall, the relatively high pollen concentration at DGN Co during the MCA is consistent with increased non-herbaceous pollen input, but its cause cannot be uniquely attributed to ASM-related hydroclimatic variability.

### 4.3. Periodic Variation in Pollen Concentration

The approximately 1.3–1.4 ka variability identified in the DGN Co total pollen concentration record is comparable in timescale to Holocene North Atlantic Bond events. These events were originally identified by recurrent increases in ice-rafted debris in North Atlantic marine sediments and have been associated with millennial-scale climate variability [31,32]. Although Bond events are often characterized by an approximately 1.5 ka recurrence interval, the mean interval between successive Holocene ice-rafting events in the VM 29-191 record was estimated to be 1374 ± 502 years (mean ± 1 SD) [32], comparable to the approximately 1.3–1.4 ka spectral peak identified in the DGN Co record. Nevertheless, this similarity provides a basis for comparison but does not, by itself, demonstrate a causal relationship or climatic teleconnection between the North Atlantic and the Asian monsoon region.

Comparable millennial-scale variability has also been reported in paleoclimate records from the Indian and East Asian monsoon regions. Leuschner and Sirocko [52] identified a periodic component near 1.45 ka in dust flux and upwelling-related proxy records from the western Arabian Sea. Similarly, sedimentary geochemical records from the northeastern Arabian Sea revealed periodicities of approximately 1425 and 1527 years [53]. At Lake Gonghai in northern China, cross-spectral analyses identified periodicities of approximately 1.4–1.5 ka in pollen and pollen-based precipitation records, while lake-sediment geochemical proxies exhibited a periodicity near 1585 years [54]. Together, these studies indicate that variability on broadly similar timescales occurs in several Asian monsoon settings. However, comparable periodicities do not necessarily imply synchronous changes or a common forcing mechanism. The DGN Co signal may therefore represent a quasi-millennial-scale feature whose relationship to these regional records remains uncertain.

**Figure 9:**
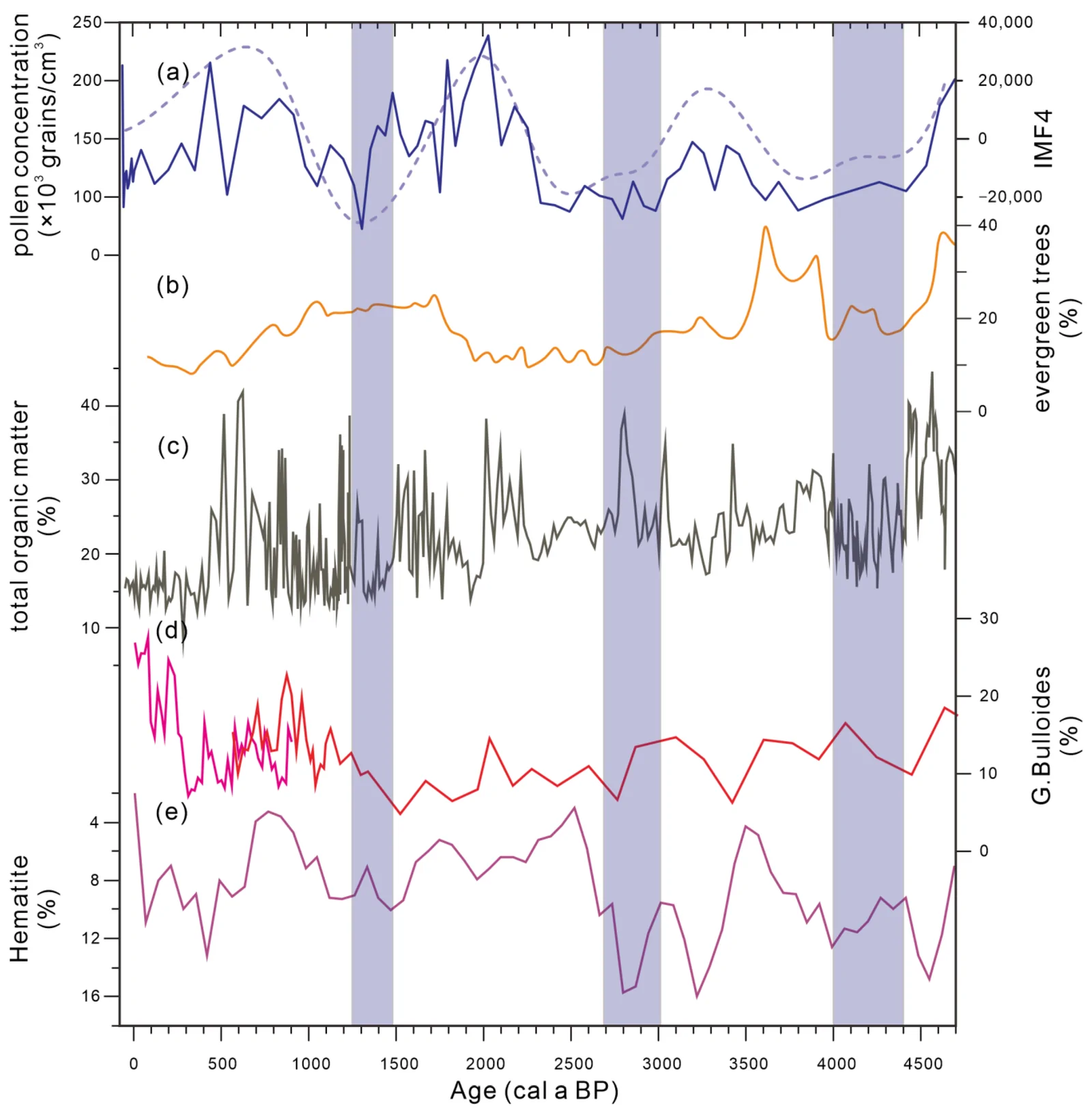
Comparison of the DGN Co total pollen concentration record with regional Asian monsoon records and North Atlantic ice-rafting variability over the past approximately 4.7 ka. (a) Total pollen concentration (solid line, left axis) and the fourth intrinsic mode function (IMF4) extracted by ensemble empirical mode decomposition (EEMD; dashed line, right axis) from DGN Co (this study). (b) Percentage of subtropical evergreen tree pollen from Lake Tianchi (Yunlong) in the Hengduan Mountains [9]. (c) Total organic matter (TOM) content of sediments from Huguangyan Maar Lake, southern China [33]. (d) Relative abundance of the planktonic foraminifer *Globigerina bulloides* in ODP Hole 723A and box core RC2730 from the Arabian Sea, reflecting variations in Indian summer monsoon-related upwelling [55]. (e) Percentage of hematite-stained grains in the North Atlantic MC52–V29-191 composite record, reflecting variations in ice-rafted debris deposition [31,32]. Shaded bands highlight selected cold intervals recurring on millennial timescales.

To place the DGN Co record in a broader context, Figure 9 compares total pollen concentration and its fourth intrinsic mode function (IMF4) with selected records of vegetation change, lake-sediment composition, monsoon-related upwelling, and North Atlantic ice-rafted debris over the past approximately 4.7 ka. The DGN Co total pollen concentration record exhibits relatively low values around 4.2, 2.8, and 1.4 ka, intervals that have also been discussed in previous studies of Asian monsoon variability in relation to millennial-scale climate events. For example, variations in monsoon-related upwelling in the Arabian Sea have been linked to North Atlantic climate changes [55]. At Huguangyan Maar Lake in southern China, decreases in cosmogenic beryllium records have been interpreted as possible expressions of Bond-like events around 4.2, 2.8, and 1.4 ka [33]. Similarly, the Dongge Cave speleothem record has also been examined for possible relationships between Asian summer monsoon variability and North Atlantic climate changes [34]. Although these comparisons provide a useful framework for interpreting the DGN Co record, apparent similarities in timing should be treated cautiously because the records differ in proxy type, chronological uncertainty, temporal resolution, and climatic interpretation.

Of these intervals, the approximately 4.2 ka event has been associated with widespread environmental changes across several regions. In the HDMs, the Lake Tianchi (Yunlong) pollen record documents a marked decline in subtropical evergreen broadleaved taxa between approximately 4.5 and 3.9 ka, interpreted as a response to cooler and drier conditions [9]. The relatively low pollen concentration at DGN Co around 4.2 ka may reflect a broadly comparable regional environmental response, although the temporal resolution of the record limits precise determination of the timing and duration of this feature. More broadly, the similarities among these records suggest a possible relationship between high-latitude climate variability and Asian monsoon dynamics during the mid- to-late Holocene. Such a relationship may have involved ocean–atmosphere interactions and changes in monsoon circulation and moisture transport. However, the available evidence is insufficient to establish a direct teleconnection or common forcing mechanism, and differences in monsoon dynamics, topography, and local environmental conditions may have contributed to spatially variable ecological responses.

Several limitations must be considered when evaluating the quasi-millennial-scale variability identified at DGN Co. The approximately 4.7 ka record contains only three to four oscillations at the inferred 1.3–1.4 ka timescale, limiting the reliability of the period estimate. Although EEMD and Lomb–Scargle analyses identified variability at similar timescales, both were applied to the same pollen concentration record and thus do not provide independent confirmation of a persistent periodicity. Moreover, interpolation onto a 25-year grid does not increase the amount of independent information provided by the original 72 samples, and interpolation of unevenly spaced paleoclimate data may introduce biases into spectral estimates [56]. The Lomb–Scargle spectral peak exceeded the local 95% significance threshold only after linear detrending and remained below the 99% threshold, based on a robust AR(1) red-noise model. Because the identification of periodic signals depends on the estimation of the background noise spectrum [57], and local spectral significance may overstate the evidence for periodicity when multiple frequencies are examined [58], these results require cautious interpretation. The sensitivity of this result to alternative red-noise assumptions has not been evaluated, and age-model uncertainty was not explicitly incorporated into the spectral analysis. These limitations constrain the statistical robustness of the inferred periodicity. Nevertheless, the identification of comparable timescales by EEMD and Lomb–Scargle analyses suggests that this variability warrants further investigation. Accordingly, the approximately 1.3–1.4 ka oscillation should be considered tentative rather than a firmly established periodicity. Higher-resolution, independently dated records, multiple climate proxies, and complementary statistical approaches will be needed to assess its robustness and explore possible links between high-latitude climate variability and Asian monsoon dynamics.

## 5. Conclusions

The pollen record from DGN Co, an alpine lake in the southern HDMs, provides valuable insights into regional arboreal pollen input and associated environmental changes over the past approximately 4.7 ka. Despite its location above the modern tree line, the pollen assemblages are dominated by arboreal taxa (89.5%), particularly *Pinus* (47.6%), indicating substantial extra-local pollen input potentially originating from forests at lower elevations. This finding highlights the importance of pollen transport in shaping high-altitude lake pollen assemblages and demonstrates their potential to provide information on vegetation beyond the immediate alpine environment.

Total pollen concentrations exhibit pronounced variations over the past 4.7 ka, with relatively low values around 4.2 ka, markedly higher values during the RWP, and relatively elevated values during the MCA. These changes may partly reflect variations in regional arboreal pollen input associated with ASM-related hydroclimatic variability. Furthermore, comparisons with other lake records from southwestern China reveal both shared patterns and regional differences, highlighting the complexity of vegetation-related environmental responses in the HDMs. Nevertheless, pollen concentration is influenced by pollen production, transport, sedimentation, and preservation, and therefore cannot be interpreted as a direct measure of forest cover or monsoon intensity.

EEMD and Lomb–Scargle spectral analyses identified a possible millennial-scale oscillatory component at approximately 1.3–1.4 ka in the total pollen concentration record. This timescale is broadly comparable to millennial-scale variability documented in North Atlantic and Asian monsoon records, providing a basis for exploring possible relationships with broader climatic fluctuations. However, the limited number of cycles, chronological uncertainties, and sensitivity of spectral significance to detrending warrant caution in interpreting this oscillation as a persistent climatic cycle or evidence of a direct climate teleconnection.

Overall, this study highlights the potential of high-altitude lake pollen records to trace long-term variations in regional arboreal pollen input and explore their possible relationships with hydroclimatic variability. The findings also emphasize the importance of considering pollen source areas and depositional processes when interpreting vegetation-related environmental signals in the topographically complex HDMs.

## Figures and Tables

**Table 1:** AMS ^14^C dating results for the DGN Co sediment core.

Lab ID	Depth (cm)	Dated Material	Conventional ^14^C Age (^14^C yr BP)	Calibrated Age Before Reservoir Correction (cal a BP, 2σ)
Bata-648624	2.5	Bulk sediment	330 ± 30	470–310
Bata-623865	18.5	Bulk sediment	1390 ± 30	1276–1350
Bata-614162	34.5	Bulk sediment	2120 ± 30	1998–2150
Bata-552047	50.5	Bulk sediment	3080 ± 30	3213–3367
Bata-614163	59.5	Bulk sediment	3600 ± 30	3831–3984
Bata-552048	67.5	Bulk sediment	4400 ± 30	4866–5048

## Data Availability

The pollen percentage and concentration data presented in this study are publicly available in Zenodo at https://doi.org/10.5281/zenodo.23319188.
